# Tumours of the Abdomen

**Published:** 1903-03-28

**Authors:** 


### Hospital Clinics and Medical Progress.

### TUMOURS OF THE ABDOMEN.

There are probably few conditions which call for greater skill in diagnosis, or which are more fertile sources of error than tumours of the abdomen. In a lecture, reported in the *Clinical Journal* of March 11th, Mr. Stanmore Bishop deals in a lucid manner with the question of recognising the various kinds that may be met with. In forming a correct opinion of the nature of any lump occurring in this region it must first be accurately located; whether it is in the abdominal wall or in the cavity contained within is the first point to be settled. If the swelling is in the abdominal wall it will become more prominent when the muscles are put into action. On the other hand, if the lump is within the cavity the stiffening of the muscles will render it less distinct. Of swellings situated in the abdominal wall itself, hernias form by far the largest class. As the openings through which they protrude occupy definite well-known positions, there is seldom much difficulty in differentiating them from all other swellings. All hernias may be usually distinguished from other lumps in the same situation by the fact that no posterior definite line of demarcation can be felt, while reducibility will confirm the diagnosis. If not reducible and containing a solid body doubt may arise, and in such a case it may be that operation alone can decide. Mural swellings other than herniæ are fibromata, lipomata, neuromata, or myomata, or are malignant or syphilitic. If the swelling is clearly marked off from the general peritoneal cavity, it has to be determined whether its contents are fluid or solid. In settling this point, puncture is never justifiable; it is almost criminal. Only in ascites is paracentesis permissible. The character of the contents of the tumour, whether solid or fluid, can be usually determined by skilful palpation. Much may be learnt by considering the mobility or otherwise of the tumour. If it moves readily with respiration it must be in close connection with the diaphragm. Hepatic and gall-bladder swellings move most of all. Gastric tumours move freely with the movements of breathing if they involve the upper part and body of the stomach, but not when they affect the pylorus. Splenic and renal swellings move downwards with forced inspiration, but not freely, and they can often be held by the examiner's hand in the position which inspiration has given them. Tumours which do not move with respiration may be visible on inspection or may require bimanual examination for their detection. If the tumour can be moved freely in any direction it is most likely to be situated in the omentum, small intestine, or mesentery, or it may be a subperitoneal fibroma or an ovarian growth, the pedicle of which has yielded. A good point in determining the seat of origin of a mobile lump is given by Mr. Bishop. The circle through which the tumour moves is noted, and the centre of this circle denotes the position of the organ from which the growth probably arises. The organ once identified, the kinds of tumour peculiar to it may be passed in review, and their differentiation effected by further observations. Perfect immobility in an abdominal growth almost always points to its having a retroperitoneal origin; in such a case, if not aneurysmal or pancreatic, it is probably sarcomatous or syphilitic. Renal tumours are usually movable to some extent. Retro-peritoneal glands are seldom sufficiently enlarged to be felt by palpation. The outline of the abdomen viewed from the side will often give valuable information. In a normal abdomen the outline moves synchronously with respiration. Should no tumour be present the movement is seen all along the line from xiphoid cartilage to pubes. Vibration is communicated along its whole length. But if a tumour is present sufficiently large to press against the abdominal wall, vibration ceases at this point. Certain swellings produce almost characteristic outlines, thus in ascites if the abdomen is not fully distended it is flattened in the centre whilst bulging in the flanks; if distension is complete, the outline is uniformly convex from xiphoid cartilage to pubes. The swelling of a pregnant uterus projects prominently forward in the centre, but the sides are depressed and the skyline is flattened near the xiphoid, but bulging out firmly at some distance below, while it rises gradually from the pubes. In fibroid disease of the uterus the rise is abrupt towards both pubic and xiphoid extremities. In this case the outline may be smooth and uniform, or irregular. The rounded even form may be distinguished from that due to an ovarian tumour by the fact that, whilst in both the rise from the pubes is abrupt, that from the xiphoid end also is abrupt in the case of fibroids, but gradual in ovarian cysts.

